# Supplementary material for: Liver-stage fate determination in Plasmodium vivax parasites: Characterization of schizont growth and hypnozoite fating from patient isolates
Source: Front Microbiol. 2022 Sep 23;13:976606. doi: 10.3389/fmicb.2022.976606 (PMC9539820; doi:10.3389/fmicb.2022.976606)
Supplement: Supplementary file 1 [file Data_Sheet_1.zip › Datasets Dictionary.DOCX]

**Experiment 1 Dataset 1.**

Raw data used for the analyses on the total number of parasites and the proportion of hypnozoites in experiment 1.

Data dictionary:

#id: individual identifier

#plate: culture plate ID

#well: plate well

#hepcells: hepatoctye donor

#spzwell: nr of sporozoite per well

#case: pv cases

#nucleicount: nr of hepatocyte nuclei per well

#nrparasites: nr of pv parasites per well

#nrschizonts: nr of schizonts per well

#nrhypno: nr of hypnozoites per well

#repwell: replicate well

#catspz: sporozoite categorical variable

**Experiment 1 Dataset 2.**

Raw data used for the analysis of the schizont size in the experiment 1.

Data dictionary:

#wells: ID plate well

#heps: a 4 level categorical variable for each type of hepatocyte

#spzwell: a continuous variable corresponding to the nr of sporozoite per well (inocula size), 8 values (12k,15k,17k,19k,21k, 24k,27k,30k)

#case: a 3 level categorical variable corresponding to the *Plasmodium vivax* cases used

#schizarea: a continuous variable corresponding to the schizont area (in µm2)

#rep: replicate well (individualized well to correct for schizonts measured in the same well) –

#cspz: an 8 level categorical variable corresponding to the nr of sporozoite per well

#plate: culture plate ID

#by the design used rep is nested in plate#

**Experiment 2**

Raw data used for the analyses on the total number of parasites and the proportion of hypnozoites in experiment 2.

Data dictionary:

#well: plate well

#nuclei: nr of hepatocyte nuclei per well

#hypno: nr of hypnozoites per well

#schizonts: nr of schizonts per well

#cell: hepatoctye donor

#spz: nr of sporozoite per well

#repwell:replicate wells

#nrpar: nr of liver stage parasites per well

**Experiment 3**

Raw data used for the analyses on the total number of parasites and the proportion of hypnozoites in experiment 3.

Data dictionary:

#assayversion: a 6-level categorical variable corresponding to the different types of assay ran

#hepage:a 3-level categorical variable corresponding to the age of hepatoctye when infected with sporozoites (D1: one day post-seeding, D2: 2 days post-seeding, D3: 3 days post-seeding)

#pvcase: a categorical variable corresponding to the plasmodium vivax cases used to infect the plate

#spzwell: a continuous variable corresponding to the nr of sporozoite per well (inoculum size)

#tot: total number of parasites per well (hypno+schizont)

#hypno:average number of hypnozoites per well

#schizont:average number of schizonts per well

#avgnrnuclei: average number of hepatocyte nuclei per well

#season: a two-level categorical variable corresponding to the dry or rainy seasons feeds

#recpv: a two-level categorical variable corresponding to a recurrent pv ('yes': already visited once the clinic) or not ('no')

#visitcon: a continuous variable corresponding to the visit number of the recurrent pv

#sex: a 2-level categorical variable corresponding to the patient sex, Male (M) or Female (F)
