# Supplementary material for: Liver-stage fate determination in Plasmodium vivax parasites: Characterization of schizont growth and hypnozoite fating from patient isolates
Source: Front Microbiol. 2022 Sep 23;13:976606. doi: 10.3389/fmicb.2022.976606 (PMC9539820; doi:10.3389/fmicb.2022.976606)
Supplement: Supplementary file 1 [file Data_Sheet_1.zip › Supplementary Figure 1.PDF]

Plate maps

Plate 1

		UBV												HHR											
	1	2	3	4	5	6	7	8	9	10	11	12	13	14	15	16	17	18	19	20	21	22	23	24	
A																									
B																									
C																									Pv case 1
D		6000	9000	12000	15000	17000	19000	21000	25000	27000	30000			6000	9000	12000	15000	17000	19000	21000	25000	27000	30000		
E																									
F																									
G																									
H																									

Plate 2

		BGW												OTW											
	1	2	3	4	5	6	7	8	9	10	11	12	13	14	15	16	17	18	19	20	21	22	23	24	
A																									
B																									
C																									Pv case 1
D		6000	9000	12000	15000	17000	19000	21000	25000	27000	30000			6000	9000	12000	15000	17000	19000	21000	25000	27000	30000		
E																									
F																									
G																									
H																									

Plate 3

		UBV												HHR											
	1	2	3	4	5	6	7	8	9	10	11	12	13	14	15	16	17	18	19	20	21	22	23	24	
A																									
B																									
C																									Pv case 2
D				12000	15000	17000	19000	21000	25000	27000	30000					12000	15000	17000	19000	21000	25000	27000	30000		
E																									
F																									
G																									
H																									
I																									
J																									
K																									Pv case 3
L		6000	9000	12000	15000	17000	19000	21000	25000	27000	30000			6000	9000	12000	15000	17000	19000	21000	25000	27000	30000		
M																									
N																									
O																									
P																									

Plate 4

		BGW												OTW											
	1	2	3	4	5	6	7	8	9	10	11	12	13	14	15	16	17	18	19	20	21	22	23	24	
A																									
B																									
C																									Pv case 2
D				12000	15000	17000	19000	21000	25000	27000	30000					12000	15000	17000	19000	21000	25000	27000	30000		
E																									
F																									
G																									
H																									
I																									
J																									
K																									Pv case 3
L		6000	9000	12000	15000	17000	19000	21000	25000	27000	30000			6000	9000	12000	15000	17000	19000	21000	25000	27000	30000		
M																									
N																									
O																									
P																									
