# Supplementary material for: Liver-stage fate determination in Plasmodium vivax parasites: Characterization of schizont growth and hypnozoite fating from patient isolates
Source: Front Microbiol. 2022 Sep 23;13:976606. doi: 10.3389/fmicb.2022.976606 (PMC9539820; doi:10.3389/fmicb.2022.976606)
Supplement: Supplementary file 1 [file Data_Sheet_1.zip › Supplementary Figures and Table legends.DOCX]

Supplementary Material

# Supplementary Figures Legends

**Supplemental Figure 1. Plate maps for Experiment 1.** PHH donor lots UBV, HHR, BGW, and OTW were seeded three days prior to infection into the same set of plates and infected with the same range of sporozoites from one of three *P. vivax* cases. Values indicate the quantify of sporozoite inoculum size used for each well. Six replicate wells were used per condition.

**Supplemental Figure 2. Liver stage parasite growth metrics from Experiment 1.** Shown are parasite size classes for each *P. vivax* case infected into each PHH donor lot. Each line represents the sporozoite inoculum size indicated in respective keys.

**Supplemental Figure 3. Relationship of net infection quantity versus schizont growth.** Each marker represents the average schizont growth area (μm^2^) in a single well seeded with the indicated PHH donor lot and infected with one of three *P. vivax* cases. PHH donor lots HHR and OTW host a fewer number of larger schizonts, while lots BGW and UBV host relatively smaller schizonts.

**Supplemental Figure 4. Analysis of the average number of *P. vivax* parasites per well from Experiment 3.** Data are aggregated from the 0.1% DMSO control wells of 132 assay plates initiated from 51 *P. vivax* cases for liver stage drug discovery and development*.* Data are categorized by (**A**) sporozoite inoculum size, (**B**) hepatocyte age at infection, (**C**) the presence of 100μM 1-aminobenzotriazole (ABT) in culture media, (**D**) the season in which the case was collected, or **(E**) the sex of the vivax patient. Asterisks indicate significant differences (Post hoc Tukey’s pairwise comparisons, ** P=0.001). Bars represent ± SE.

**Supplemental Figure 5. Analysis of the average proportion of *P. vivax* hypnozoites per well from Experiment 3.** Data are aggregated from the 0.1% DMSO control wells of 132 assay plates initiated from 51 *P. vivax* cases for liver stage drug discovery and development*.* Data are categorized by (**A**) sporozoite inoculum size, (**B**) hepatocyte age at infection, (**C**) the presence of 100μM 1-aminobenzotriazole (ABT) in culture media, (**D**) the season in which the case was collected, **(E**) the sex of the vivax patient, or (**F**) the number of visits to a malaria clinic an individual vivax patient experienced. Asterisks indicate significant differences (Post hoc Tukey’s pairwise comparisons * P<0.05). Bars represent ± 95% CI.

# Supplementary Table Legend

**Supplemental Table 1. Primary human hepatocyte donor lot quality control data including donor life history and metabolism.** Data were drawn from the Certificate of Analysis from the PHH donor lot vendor, BioIVT, used with permission. ND: Not determined. BQL: below quantifiable limit.
