# Supplementary figures and images for: Liver-stage fate determination in Plasmodium vivax parasites: Characterization of schizont growth and hypnozoite fating from patient isolates
Source: Front Microbiol. 2022 Sep 23;13:976606. doi: 10.3389/fmicb.2022.976606 (PMC9539820; doi:10.3389/fmicb.2022.976606)

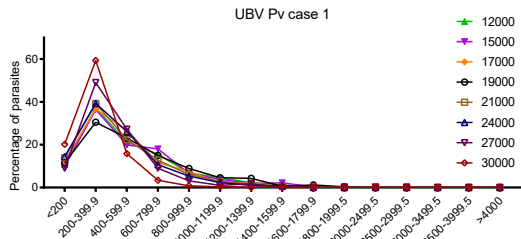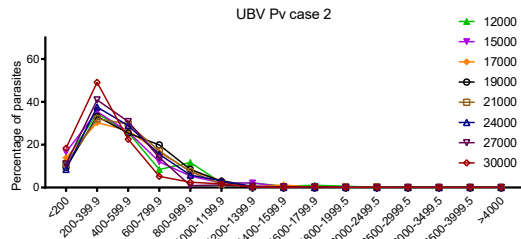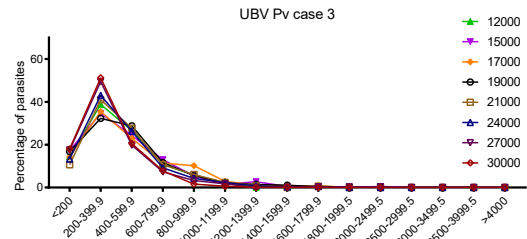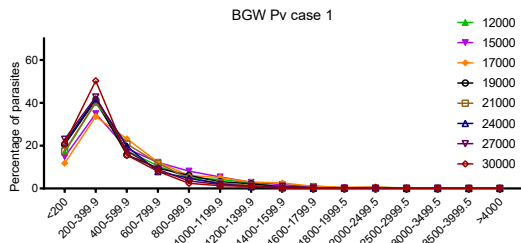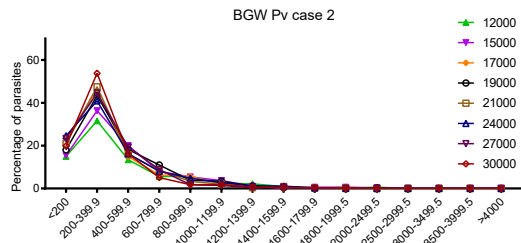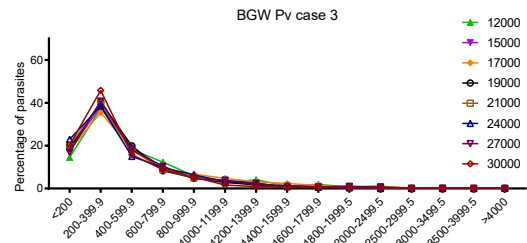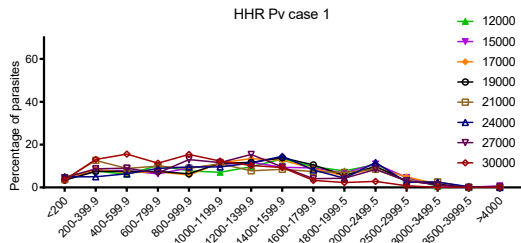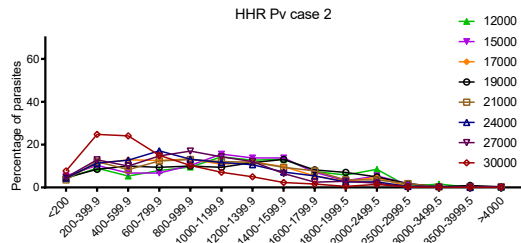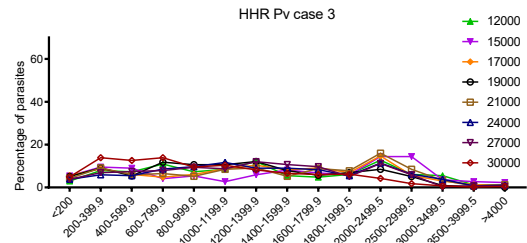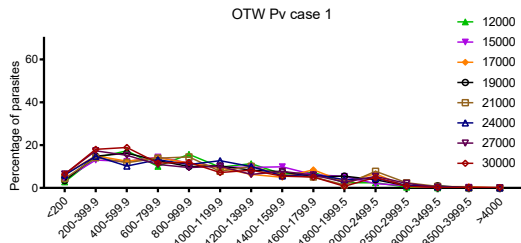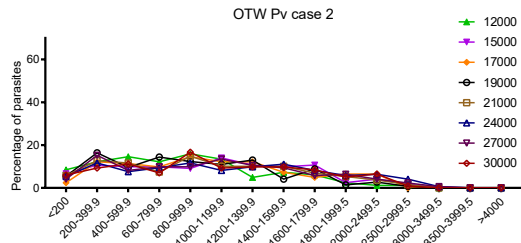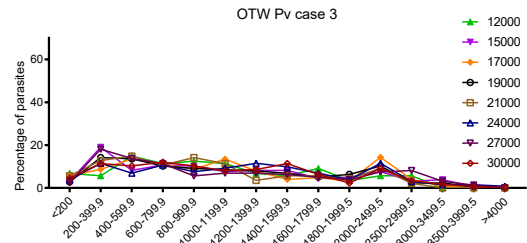

Supplement: Supplementary file 1 [file Data_Sheet_1.zip › Supplementary Figure 2.PDF]

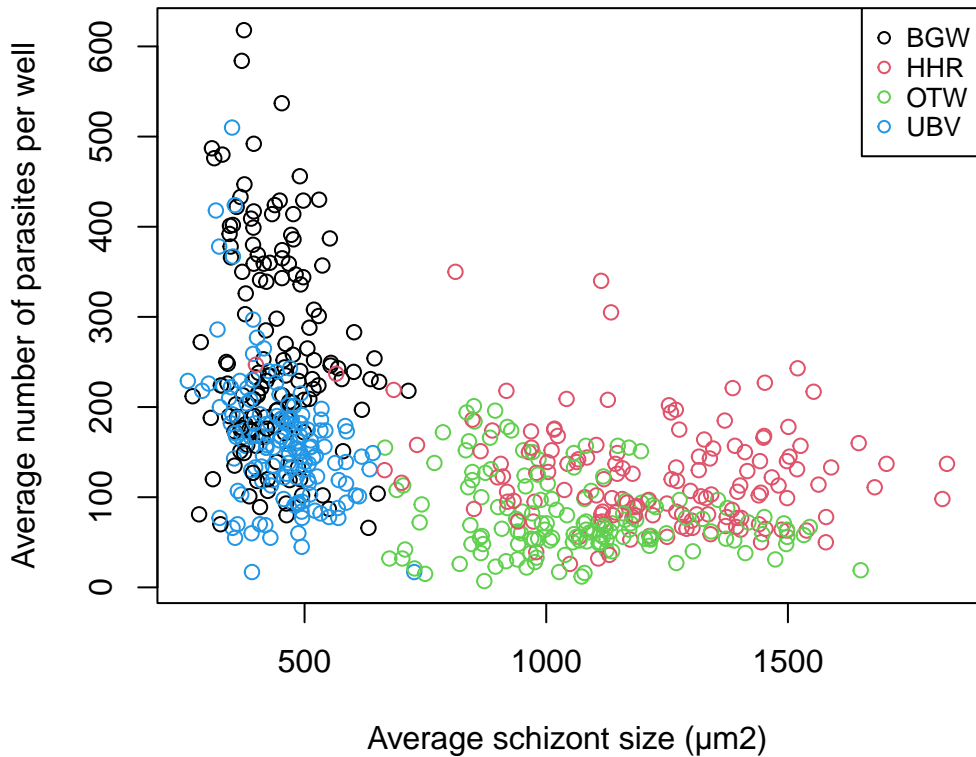

Supplement: Supplementary file 1 [file Data_Sheet_1.zip › Supplementary Figure 3.PDF]

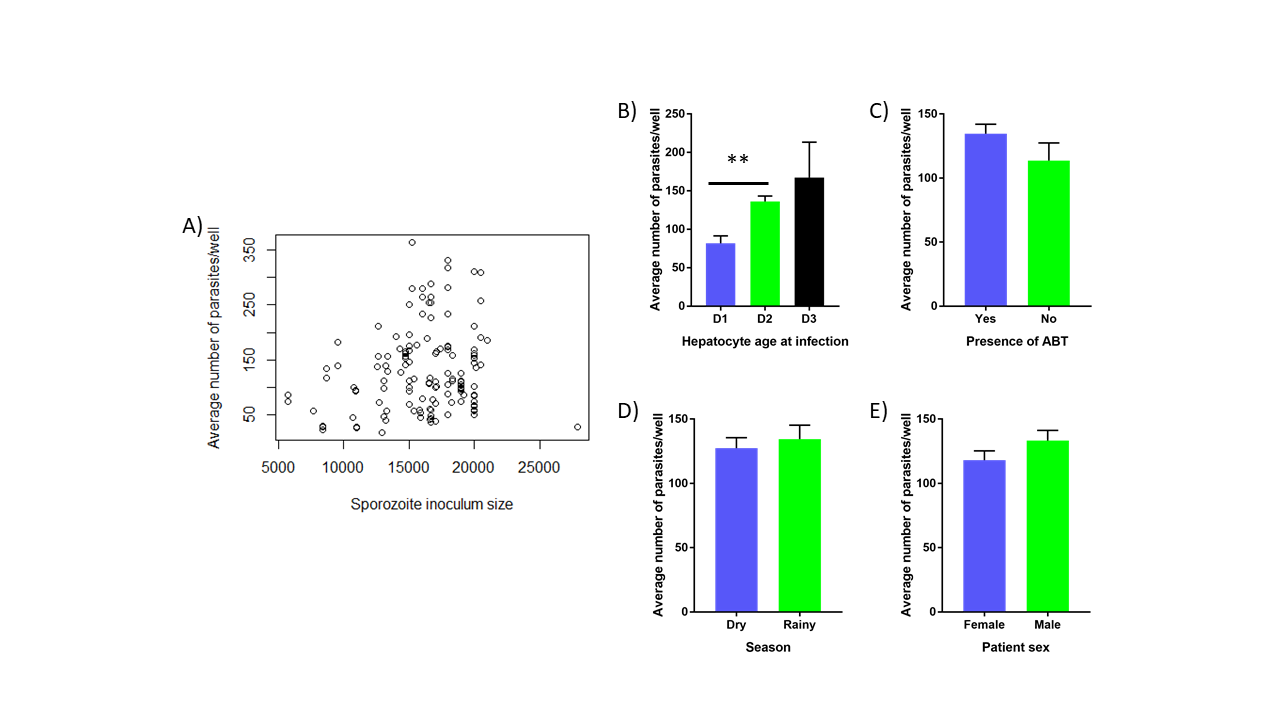

Supplement: Supplementary file 1 [file Data_Sheet_1.zip › Supplementary Figure 4.TIF]

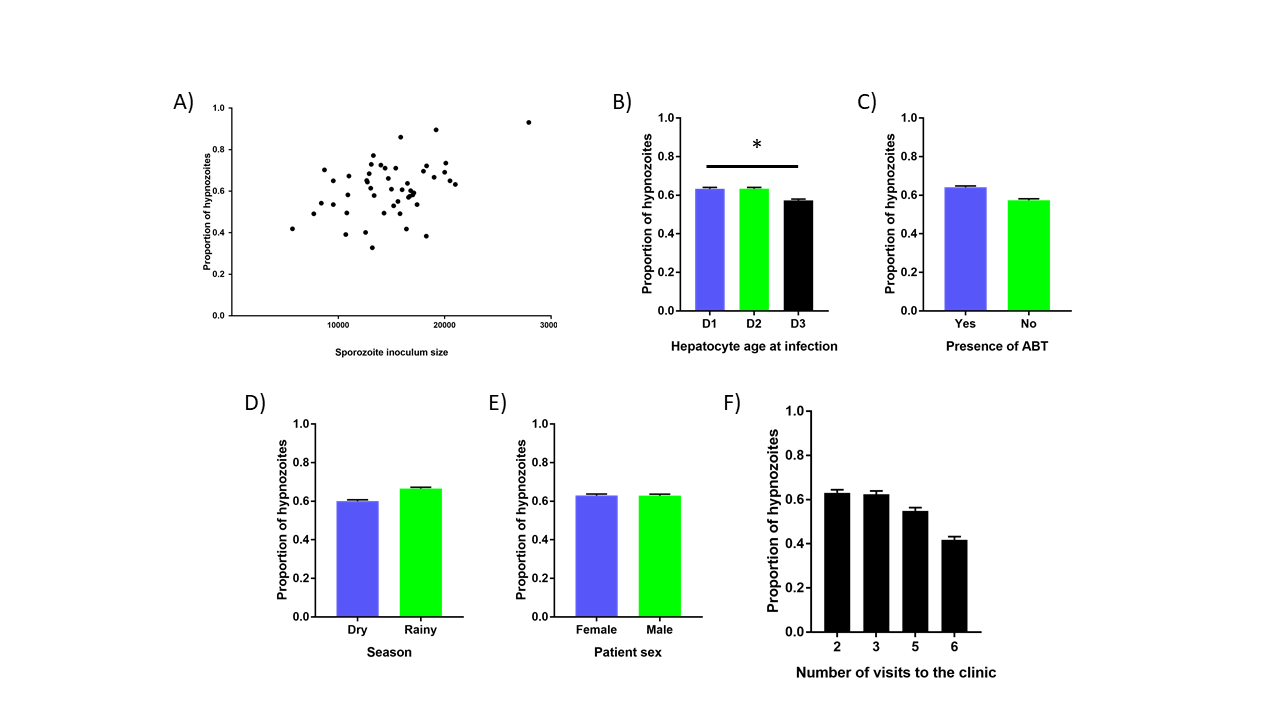

Supplement: Supplementary file 1 [file Data_Sheet_1.zip › Supplementary Figure 5.TIF]
